# Post-Intensive Care Syndrome after Critical Illness: An Imperative for Effective Prevention

**DOI:** 10.3390/jcm11206203

**Published:** 2022-10-21

**Authors:** Nicolas Paul, Björn Weiss

**Affiliations:** Department of Anesthesiology and Operative Intensive Care Medicine, Campus Charité Mitte and Campus Virchow-Klinikum, Charité—Universitätsmedizin Berlin, Corporate Member of Freie Universität Berlin and Humboldt-Universität zu Berlin, Augustenburger Platz 1, 13353 Berlin, Germany

Over the last decades, the importance of intensive care has considerably increased. In the US for example, the number of intensive care unit (ICU) beds has grown by 15% between 2000 and 2009 [1], and costs of intensive care have increased by 44.2% between 2000 and 2005 [2]. Despite the fact that today’s ICU patients are more often of advanced age and more severely ill [3], mortality rates have been steadily declining [4], which has been attributed to advances in research and technology.

Research in intensive care has traditionally aimed at reducing short-term mortality [5,6], but we have learned that the growing cohort of ICU survivors frequently faces long-term sequelae, which may have more importance to patients and families than mere survival [7,8]. These sequelae include reduced cognitive functions [9], impairments of mental health (particularly depression [10], post-traumatic stress disorder [11], and anxiety [12]), and reduced physical function [13], commonly summarized as post-intensive care syndrome (PICS) [14]. Impairments may persist years after ICU discharge [13], and are accompanied by a reduced health-related quality of life [15].

Despite the sound research foundation on PICS epidemiology, knowledge of effective treatment strategies is limited [16]. Interventions targeting cognitive function, mental health, or physical function yield inconclusive efficacy [17]. Care after ICU discharge is often suffering from little coordination and fragmentation [18]. Coordinating care in post-ICU recovery centers has been considered an important element of PICS treatment, but thus far lacked robust efficacy data [17]. A Cochrane review from 2018 concluded that there is insufficient evidence to determine the effectiveness of ICU follow-up services [19].

With limited effective treatment options for already manifest PICS currently at our disposal [17], the focus should be put on PICS prevention and risk factor mitigation during ICU treatment. To this end, strategies to limit deep and long sedation, to avoid the use of benzodiazepines, and to manage delirium may reduce cognitive impairments; strategies to optimize nutritional intake and early mobilization may improve physical function; and early family engagement may improve the mental health of ICU survivors [20,21,22].

The Special Issue *Enhancing Recovery after Intensive Care Medicine in Clinical Practice* in the *Journal of Clinical Medicine* adds important aspects to potential ways to prevent PICS by advancing evidence-based treatment in the ICU. In a prospective single-center study, Duda and Krzych showed that plasma neutrophil gelatinase-associated lipocalin, an acute-phase protein, predicts ICU and hospital mortality [23]. Predictive performance may be increased if combined with the disease severity score APACHE II.

With respect to cognition, two articles explored sedation practices. In a retrospective single-center study, Weiss et al. compared 3314 mechanically ventilated ICU patients that received either the intravenous benzodiazepines lormetazepam or midazolam with respect to survival and sedation characteristics [24]. Without adjusting for sedation intensity, the use of midazolam, which has been connected to prolonged deep sedation, was associated with increased in-hospital mortality. When adjusting for sedation intensity, the survival benefit of lormetazepam disappeared. The authors concluded that lormetazepam may be more suitable than midazolam to achieve light sedation targets. In another retrospective single-center study, Weiss et al. compared 662 matched patient pairs undergoing cardiac surgery who either received propofol or etomidate as an induction agent [25]. The authors found that postoperative sepsis was not associated with a single dose of etomidate, but patients receiving etomidate showed higher rates of hospital-acquired pneumonia compared to patients receiving propofol.

With respect to physical function, ICU-acquired weakness has been identified as a key factor in hampering recovery [21]. As the female sex is considered a risk factor for ICU-acquired weakness, Engelhardt et al. performed a secondary, sex-specific analysis of two trials on skeletal muscle metabolism in mechanically ventilated ICU patients [26]. While the prevalence of ICU-acquired weakness did not differ between male and female sex, female sex was associated with a significantly reduced insulin sensitivity index and lower myocyte cross-sectional area. In another study on neurological disorders that may contribute to physical impairments after ICU discharge, Totzeck et al. explored the safety and diagnostic potential of total plasma exchange among 20 ICU patients with neuromuscular junction disorders with uncertain disease activity or diagnosis [27]. To identify patients prone to long-term physical impairment, Moayed et al. developed a prediction model for physical disability after discharge in Iranian ICU patients [28]. Educational level lower than elementary school, inability to sit without support, and having a fracture were significantly associated with worsening physical function after discharge.

With respect to mental health, Krampe et al. conducted a systematic review of patient stressors in the ICU [29]. They identified 137 stressor items in four domains, namely physical, treatment, and disease-related stressors (e.g., being in pain), mental health stressors (e.g., fear of death), communication stressors (e.g., not being able to talk), and environmental stressors (e.g., hearing other patients cry out). Addressing the most severe stressors may contribute to better mental health outcomes after discharge.

In summary, this Special Issue added small bricks of evidence on the prevention of PICS-related impairments of cognition, mental health, and physical function by optimizing treatment in the ICU. Additional high-quality studies on PICS prevention and, more importantly, interventions to mitigate PICS after ICU discharge are urgently needed. 

We would like to express our gratitude to the contributing authors for their excellent scientific input, to the reviewers and co-editors for their perceptive comments on the submissions, and, lastly, to the *Journal of Clinical Medicine* for their administrative assistance.
